# A Decade of Electrical Injuries: An Epidemiological Analysis of Emergency Department Data

**DOI:** 10.1155/emmi/1146087

**Published:** 2025-07-18

**Authors:** Yasemin Adalı, İbrahim Türkçüer, Yasemin Berberoğlu, Veli Kaan Aydın, Atakan Yılmaz, Mert Özen, Murat Seyit, Alten Oskay, Aylin Köseler

**Affiliations:** ^1^Centre for Public Health, School of Medicine, Dentistry, and Biomedical Sciences, Queen's University Belfast, Belfast, UK; ^2^Department of Biophysics, Pamukkale University Faculty of Medicine, Denizli, Turkey; ^3^Department of Emergency Medicine, Pamukkale University Faculty of Medicine, Denizli, Turkey

**Keywords:** electrical injury, epidemiology, high-voltage injuries, low-voltage injuries

## Abstract

**Objective:** Electrical injuries present a diagnostic and management challenge due to their diverse clinical manifestations and potential complications. Although the current guidelines recommend cardiac monitoring in selected cases, the criteria for risk stratification remain limited. This study aimed to evaluate the epidemiological and clinical characteristics of patients with electrical injuries admitted to the emergency department over a 10-year period.

**Methods:** This retrospective study reviewed medical records of patients admitted to the Pamukkale University Hospital between 2014 and 2024 due to electrical injuries. Data collected included age, sex, time of injury, voltage level, current type and source, contact site, transthoracic current pathway, ECG findings, laboratory results (troponin T, CK-MB, and potassium), work-related status, and mortality. The primary outcomes were epidemiological characteristics, ECG abnormalities, and laboratory evidence of myocardial injury.

**Results:** A total of 112 patients were identified; 91 (81%) patients were male, with a mean age of 31.8 years. High-voltage injuries (> 1000 V) occurred in 10 patients, while low-voltage injuries (< 1000 V) were seen in 80 (60.6%) patients. Work-related injuries accounted for 14.3% of cases. One patient died due to trauma following high-voltage exposure. Troponin T was elevated in 57 of 92 tested patients (62.0%), CK-MB in 25 (22.3%), and hyperkalemia in six (5.3%). ECG abnormalities were detected in 16 patients (14.3%).

**Conclusion:** Biochemical evidence of myocardial injury was observed even in low-voltage exposures, raising concerns about the safety of early discharge based solely on clinical presentation and ECG findings. Prospective studies are needed to refine risk assessment strategies in electrical injury cases.

**Summary**

• Elevated troponin (62%) and CK-MB (22.3%) levels were more frequently observed in low-voltage electrical injury cases than expected.
• Conscious patients with low-voltage exposure and normal ECG may still be at risk of myocardial injury and require careful monitoring.
• High-sensitivity cardiac troponin testing should be considered during initial assessment to better identify subclinical myocardial damage.

## 1. Introduction

Electrical injuries can result in a wide range of clinical consequences, from minor burns to life-threatening conditions such as myocardial infarction, cardiomyopathy, heart failure, respiratory distress, and cardiac arrest, depending on the voltage level and current pathway involved [1]. Due to the heterogeneous nature of both high- and low-voltage injuries, postdiagnosis care often requires a multidisciplinary approach, involving cardiology, emergency medicine, and sometimes surgical teams [2].

Most electrical injuries are caused by low-voltage currents, and critical factors such as the source of electricity, entry and exit points, and current pathway play an essential role in determining the severity of internal injury [3]. Tissue damage results from the interaction between exposure duration, voltage, and tissue resistance [4]. Current that travels through the thorax, particularly transthoracic pathways, carries a greater risk for cardiac involvement, including conduction abnormalities and myocardial damage [5].

Despite the potential severity, comprehensive epidemiological data on electrical injuries remain scarce worldwide [6]. Most are attributed to standard household voltage levels (100–240 V), and injuries are typically classified as low voltage (< 1000 V) or high voltage (> 1000 V) [3]. While low-voltage injuries are often asymptomatic or minimally symptomatic, they can still pose a risk for sudden cardiac death, warranting careful evaluation [7, 8]. Consequently, prevention remains the most effective strategy to reduce both the incidence and severity of such injuries.

The present study aimed to evaluate the incidence and frequency of cardiac complications, including arrhythmias and mortality, among patients presenting to the emergency department with electrical injuries over a 10-year period.

## 2. Materials and Methods

### 2.1. Study Design and Population

This retrospective, single-center study was conducted at the Emergency Department of the Pamukkale University Faculty of Medicine Hospital. Medical records of all patients admitted with electrical injuries between January 1, 2014, and January 1, 2024, were reviewed. The study protocol was approved by the Pamukkale University Medical Ethics Committee (Approval number: E-60116787-020-526509) and carried out in accordance with the ethical principles outlined in the Declaration of Helsinki.

The Pamukkale University Hospital is a tertiary care center with 853 inpatient beds and receives approximately 1.7 million outpatient visits annually. All patients presenting with electrical injuries during the 10-year study period were eligible for inclusion. No age or sex restrictions were applied. The primary aim of the study was to evaluate the epidemiological characteristics of these patients, as well as the frequency of arrhythmias and abnormal cardiac-related laboratory parameters, such as elevated biomarkers indicative of myocardial stress or injury, following electrical exposure.

### 2.2. Data Collection

All patients with electrical injuries admitted to the Pamukkale University Hospital over a 10-year period were evaluated for inclusion in this study. To ensure comprehensive case identification, a systematic keyword and ICD-10 code search was performed using the hospital's electronic medical record system (Probel). Keywords included “electrical injury,” “electrical accident,” “high-voltage injury,” “low-voltage injury,” and “lightning.” The following ICD-10 codes were used to capture relevant cases: W85 (exposure to electric transmission lines), W86 (exposure to other specified electric current), W87 (exposure to unspecified electric current), W29 (contact with powered hand tools and household machinery), T75.4 (effects of electric current), and T75.0 (effects of lightning) [9].

Data collected from the electronic system included details related to the injury event: time of incident, voltage level, type of current (AC or DC), point and location of contact, and presence of a transthoracic current pathway. Additional variables included the current source, whether the event was work-related, diagnosis on admission, prehospital status, and mode of transport to the emergency department. Information regarding prehospital interventions and loss of consciousness was also retrieved.

Demographic and clinical characteristics such as age, sex, triage category (Glasgow Coma Scale), initial complaints, ECG findings, and associated secondary injuries were recorded. Laboratory parameters included serum levels of creatinine, CK-MB, sodium, troponin T, potassium, and calcium. In some cases, additional laboratory tests were requested at the discretion of the attending physician.

### 2.3. Data Management and Statistical Analysis

All data were handled in accordance with national data protection regulations, and patient information was anonymized before being accessed by the study coordinator. Data collection was performed by the research team (Y.A., İ.T., A.K., V.K.A., A.Y., Y.B., M.Ö., A.O., and M.S.) in two stages using Microsoft Excel. Statistical analysis was conducted using StataIC 16.1 (64 bit) (StataCorp, 4905 Lakeway Drive, College Station, TX, USA). Descriptive statistics were presented as means and standard deviations for continuous variables. Group comparisons were performed using Student's *t*-test, Chi-square test (*χ*^2^), or the Kruskal–Wallis test, as appropriate, depending on variable distribution and measurement level. A two-sided *p* value of < 0.05 was considered statistically significant. No power analysis was conducted due to the exploratory and retrospective nature of the study.

## 3. Results

**Figure 1:**
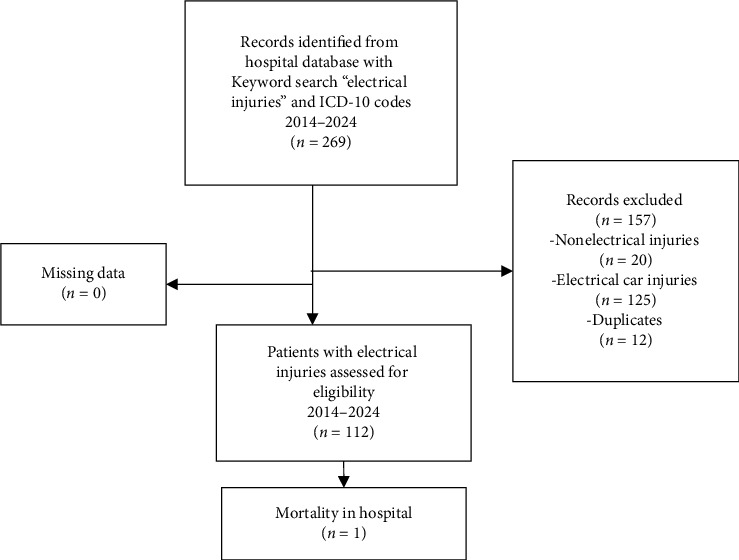
Flow diagram illustrating the selection of patients with electrical injuries in the emergency department.

A total of 269 patient records were identified through the keyword search (“electrical injury”) and relevant ICD-10 codes in the Pamukkale University Hospital electronic database for the period 2014–2024. Among these, 157 cases were excluded as they were related to electrical vehicle accidents or injuries not involving actual electrical shock. The remaining 112 patients with confirmed electrical shock injuries were included in the study cohort and had presented to the Emergency Department. Regarding mortality, only one patient, who was exposed to a high-voltage electrical current, died in the emergency department due to trauma-related complications (Figure 1).

### 3.1. Patient Characteristics

Of the 112 patients with electrical injuries in this study, 81.3% (*n* = 91) were male, and high-voltage electric shock was only observed in males. The overall average age was 31.8 ± 15.26 for all patients. The age of patients with high-voltage electrical injuries was 46.07 ± 16.33, while the age of patients with low-voltage electrical injuries was 29.8 ± 13.11. In general, it was observed that the age range of all electrical injury patients was 18–25 years (*n* = 32, 28.6%). As a seasonal change, it was observed that electrical injuries occurred in the winter months in all patients (*n* = 38, 33.9%). Additionally, 66.1% (*n* = 74) of the patients came to the emergency department self-referentially, and 63.71% of the patients had low-voltage current injuries (*n* = 51, 63.71). The most common complaint type was extremity pain (*n* = 18, 16.1%), followed by chest pain (*n* = 17, 15.2%). It was observed in 16.3% (*n* = 13) of patients injured by low-voltage electrical current had chest pain (Table 1).

As a source of electrical injuries, it was observed that all patients (*n* = 24, 21.4%) and patients with low-voltage electrical injuries (30%) were mostly caused by electric appliances. High-voltage line electricity constitutes 90% (*n* = 9) of high-voltage electrical injuries. In terms of voltage, 220 V constituted 45% (*n* = 51) of all electrical injury patients, while all patients with high-voltage injuries were over 30.000 V. About 31.2% (*n* = 25) of low-voltage electrical injuries from the patient cohort were exposed to 380 V electric current. It was observed that 21.4% (*n* = 24) of the patients were exposed to transthoracic flow. Furthermore, the left hand was the most frequently reported site of electrical entry (*n* = 19, 16.9%), followed by the right hand (*n* = 14, 12.5%). Details regarding the source of electrocution and the current pathway are summarized in Table 2.

### 3.2. Pathological Outcomes

The factors affecting the clinical features of the electrical injuries are presented in Table 3. ECG results have shown that there were 16 (14.3%) abnormal results in all patients. Furthermore, 11 (13.7%) abnormal ECG results were in the group of low-voltage electrical injury patients. Blood samples were drawn approximately 80% of the patients. Creatinine level was measured in 96 patients and was found to be elevated in five (4.5%) patients and decreased in 18 (16.1%) patients. Furthermore, troponin T was measured in 92 patients, of whom 57 (62.0%) showed elevated values. Among those with elevated troponin levels, 45 patients (78.9%) had sustained low-voltage electrical injuries. The CK-MB level was tested in 82.1% of all patients and was elevated to 25 (22.3%). Sodium calcium and potassium levels in all patients with electrical injuries were normal. The exact length of stay in the emergency department did not exceed 26 h for patients with low-voltage and high-voltage injuries. This was because patients with high voltage were referred to burn or orthopedic units.

Troponin T levels and ECG findings were analyzed across voltage exposure groups. A statistically significant association was observed between voltage level and elevated troponin levels (Table 4). The highest proportions of elevated troponin were seen in the > 1000 V group (60.0%) and the 360–750 V group (55.2%). No statistically significant association was found between ECG findings and voltage exposure (Table 4). Abnormal ECG findings were slightly more frequent in the > 1000 V group (30.0%) compared to other voltage groups, but this difference did not reach statistical significance. A strong and statistically significant association was found between troponin T levels and ECG findings (Table 5). Among patients with elevated troponin, 22.8% had abnormal ECG findings, while 77.2% had normal ECGs. Conversely, among patients with normal troponin levels, only 5.7% had abnormal ECGs.

## 4. Discussion

This analysis presents demographic, clinical, and pathological data from a cohort of patients treated in the emergency department after electrical injuries. No deaths or immediate cardiac complications were observed following low-voltage injuries, except for one patient who died as a result of head trauma complications following the accident due to fall from height after high-voltage electrical injuries. Although the number of low-voltage electrical injuries is high, it is estimated that the actual number is higher because of the lack of symptoms and not seeing a doctor in the absence of symptoms [1]. The results indicated that 16% of electrical injuries were due to work accidents. Situations that are legally followed up, such as work accidents, are recorded to pay compensation or avoid problems in the future. The lack of asymptomatic low-voltage electrical injuries outside the context of work accidents may have created selection bias in the analyses.

There is no single standard procedure for the treatment of patients with electrical injuries in Türkiye. Even with asymptomatic symptoms, cardiac arrhythmia may occur after electrical injuries; therefore, these patients are hospitalized. According to European guidelines, it has been observed that cardiac monitoring is recommended in the presence of cardiovascular, unconsciousness, cardiac arrest, and ECG abnormalities after electrical injuries [10]. In Türkiye, it has been observed that females are more likely to apply to the emergency room for injuries due to electrical injuries than in other countries [11]. In a review analysis conducted by Shih et al. in 2017, 93.9% of all patients with electrical injuries were male and 6.1% were female. Study results showed that 18.8% of females were injured after electric shock [2]. The main reason for this may be a lack of sufficient knowledge and control regarding the use of electricity.

The usefulness of laboratory parameters for electrical injuries is controversial [9, 12, 13]. In a retrospective study conducted by Ahmed et al., troponin and CK values were high in only three out of 465 patients and six out of 465 patients, respectively. The results showed that troponin levels were high in 57/112 patients, whereas CK-MB values were high in 25/112 patients [13]. Another study conducted by Pawlik et al. found high troponin values in only one out of 240 patients [6]. However, the examination of troponin and CK-MB levels among the studies conducted is insufficient. Moreover, in a retrospective study by Kopp et al., 42 patients treated for burns after electrical injuries were examined for CK serum levels, and analysis of the data revealed an association between strongly elevated CK levels and patients' risk of limb amputation and death [14]. Studies should be conducted to evaluate the risk factors for electrical injuries, and whether laboratory parameters help in risk estimation should be examined. In another study conducted with 785 patients with electrical injuries, 17 out of 533 patients (3.2%) who underwent blood tests taken had elevated troponin at baseline [9]. The sensitivity of a positive troponin test at baseline for predicting a major adverse cardiac event was found to be 83.3%, while the positive probability ratios were 36.6% and the negative predictive value was 99.9% [9]. These results suggest that troponin levels are a predictive marker of the risk of a major adverse cardiac event and it has been suggested that troponin levels should be considered in high-risk patients.

This was a single-center, retrospective study; therefore, the findings may not be fully generalizable to other populations or healthcare systems. Although data documentation in the hospital information system (Probel) was consistent for laboratory and ECG results, some patients' clinical histories were incomplete, particularly regarding the mechanism, pathway, or source of electrical injury. In addition, voltage exposure was unknown in 20 patients (17.9%), and ECG was not performed in 14 patients (12.5%), which may have affected subgroup analyses. The phase type (single-phase vs. three-phase) was not routinely recorded in medical files, limiting further stratification. Some severe cases particularly those with extensive burns may have been transferred directly to the burn unit, bypassing the emergency department, and therefore excluded from this study. Likewise, patients who died at the scene were not captured, leading to potential underestimation of more severe injury outcomes. There is also a possibility that mild electrical injuries presenting only to outpatient clinics or not documented accurately in the electronic system were missed, which may have introduced selection bias. Lastly, the type of troponin assay used may have changed over the 10-year study period, potentially influencing the comparability of results across time. Moreover, the absence of follow-up data precluded assessment of long-term clinical outcomes in patients with elevated troponin levels. Therefore, we could not determine whether biomarker elevation corresponded to actual myocardial damage or adverse cardiovascular events.

The present study demonstrated a significant association between voltage exposure and elevated troponin T levels, with higher voltages (i.e., > 1000 V and 360–750 V) more frequently resulting in biochemical evidence of myocardial injury. This finding supports the hypothesis that electrical current intensity may correlate with the degree of myocardial cell stress or damage. However, this relationship was not observed for ECG abnormalities, which showed no significant variation across voltage categories. This suggests that while voltage level may influence subclinical myocardial injury as reflected by biomarker elevation, it does not necessarily manifest as electrical conduction abnormalities detectable on ECG. Furthermore, we observed a strong association between elevated troponin T levels and abnormal ECG findings. Approximately one in four patients with elevated troponin had abnormal ECGs, while the majority had normal ECGs despite biochemical evidence of injury. This finding underscores the limited sensitivity of ECG in detecting early or mild myocardial injury in the context of electrical trauma and supports the complementary role of cardiac biomarkers in risk stratification. Collectively, these findings suggest that troponin elevation may serve as a more sensitive early indicator of cardiac involvement in electrical injury than ECG findings alone. Routine troponin testing, particularly in patients exposed to high-voltage electrical currents, may aid in identifying patients at risk of subclinical cardiac injury, even in the absence of ECG changes. However, as long-term cardiac outcomes were not available in our study, this finding should be interpreted cautiously and should not be used to guide clinical practice without further validation. Prospective studies with follow-up are needed to determine the prognostic value of troponin elevation in this setting.

## 5. Conclusion

In conclusion, the literature indicates that the low-voltage group had significantly higher troponin and creatinine values. The risk of significant adverse cardiac events is highly variable in patients with electrical injuries. The findings obtained from this study did not support the notion that conscious patients admitted with a normal ECG following a low-voltage injury could be released safely following a brief clinical evaluation that included ECG. It is recommended that an initial electrocardiogram, physical examination, and cardiac enzyme assessment, including high-sensitivity cardiac troponin, be performed to evaluate myocardial injury even in patients presenting with low-voltage electrocardiographic findings.

## Figures and Tables

**Table 1:** Patient characteristics an electrical injury information.

Variables	All electrical injuries (*n* = 112)	High voltages (*n* = 10)	Low voltage (*n* = 80)	Unknown voltage (*n* = 22)	*p* value
*Gender*					
Male *n* (%)	91 (81.25)	10 (10.9)	63 (69.2)	18 (19.8)	0.267^a^
Female *n* (%)	21 (18.75)	0	17 (80.9)	4 (19.1)	0.109^b^

*Age (mean ± SD)*	31.8 ± 15.26	46.07 ± 16.33	29.81 ± 13.11	32.47 ± 18.9	0.005^a^

*Age category*					
0–18	16 (14.3)	0	12 (15)	4 (18.2)	0.008^a^ 0.001^b^
18–25	32 (28.6)	1 (10)	25 (31.2)	6 (27.3)	
25–35	29 (25.9)	3 (30)	20 (25)	6 (27.3)	
35–45	14 (12.5)	0	14 (17.6)	0	
45–65	17 (15.2)	4 (40)	8 (10)	5 (22.7)	
65 >	4 (3.5)	2 (20)	1 (1.2)	1 (4.5)	

*Season*					
Spring	20 (17.9)	3 (30)	14 (17.5)	3 (13.6)	0.398^a^ 0.231^b^
Summer	24 (21.4)	9 (40.9)	3 (30)	26 (32.5)	
Autumn	30 (26.8)	7 (31.8)	4 (40)	19 (23.7)	
Winter	38 (33.9)	3 (13.6)	0	21 (26.2)	

*Method of arrival*					
Self-referral *n* (%)	74 (66.1)	2 (20)	51 (63.7)	21 (95.4)	0.001^a^ 0.008^b^
Ambulance *n* (%)	10 (8.9)	3 (30)	7 (8.8)	0	
Referral from other hospitals	13 (11.6)	4 (40)	9 (11.2)	0	
Unknown *n* (%)	15 (13.4)	1 (10)	13 (16.3)	1 (4.6)	

*Complaints*					
Extremity pain *n* (%)	18 (16.1)	0	11 (13.8)	7 (31.8)	0.194^a^ 0.176^b^
Chest pain *n* (%)	17 (15.2)	0	13 (16.3)	4 (18.4)	
Skin lesions *n* (%)	7 (6.2)	1 (10)	5 (6.3)	1 (4.5)	
Fainting *n* (%)	6 (5.4)	1 (10)	4 (5)	1 (4.5)	
Vomit *n* (%)	2 (1.8)	0	1 (1.2)	1 (4.5)	
Dyspnea *n* (%)	1 (0.9)	0	1 (1.2)	0	
Paralysis *n* (%)	1 (0.9)	1 (10)	0	0	
Paresthesia *n* (%)	1 (0.9)	0	1 (1.2)	0	
Others *n* (%)	3 (2.6)	0	2 (2.5)	1 (4.5)	
Unknown *n* (%)	56 (50)	7 (70)	42 (52.5)	7 (31.8)	

*Burns*					
Yes *n* (%)	23 (20.6)	7 (70)	16 (20)	0	0.001^a^ 0.003^b^
No *n* (%)	8 (7.1)	0	7 (8.8)	1 (4.5)	
Unknown *n* (%)	81 (72.3)	3 (30)	57 (71.3)	21 (95.4)	

^a^Analysis conducted with unknown variables for voltage status.

^b^Analysis conducted without unknown variables for voltage status.

**Table 2:** Source of electrocution and route of the electric current in patients.

Variables	All electrical injuries (*n* = 112)	High voltages (*n* = 10)	Low voltage (*n* = 80)	Unknown voltage (*n* = 22)	*p* value
*Current source*					
Light bulb *n* (%)	4 (3.6)	0	4 (5)	0	0.001^a^ 0.001^b^
Electric appliance *n* (%)	24 (21.4)	0	24 (30)	0	
Electrical fuse *n* (%)	1 (0.9)	0	1 (1.3)	0	
Occupational accident *n* (%)	16 (14.3)	0	16 (20)	0	
Generator *n* (%)	1 (0.9)	0	1 (1.3)	0	
Cable *n* (%)	2 (1.8)	0	2 (2.5)	0	
Electric socket *n* (%)	18 (16)	0	18 (22.5)	0	
Mains electricity *n* (%)	2 (1.8)	0	2 (2.5)	0	
Industrial electric *n* (%)	5 (4.4)	0	5 (6.2)	0	
Construction site electric *n* (%)	3 (2.7)	0	3 (3.7)	0	
Transformer electric *n* (%)	1 (0.9)	1 (10)	0	0	
High-voltage line electric *n* (%)	9 (8.1)	9 (90)	0	0	
Unknown *n* (%)	26 (23.2)	0	4 (5)	0	

*Volt*					
220 V *n* (%)	51 (45.6)	0	51 (63.7)	0	0.001^a^ 0.001^b^
320 V *n* (%)	1 (0.9)	0	1 (1.2)	0	
360 V *n* (%)	2 (1.8)	0	2 (2.5)	0	
380 V *n* (%)	25 (22.3)	0	25 (31.2)	0	
750 V *n* (%)	1 (0.9)	0	1 (1.2)	0	
300,000 V *n* (%)	10 (8.9)	10 (100)	0	0	
Unknown *n* (%)	22 (19.6)	0	0	22 (100)	

*Transthoracic flow*					
Yes *n* (%)	24 (21.4)	9 (90)	14 (17.5)	1 (4.5)	0.001^a^ 0.001^b^
No *n* (%)	31 (27.7)	0	26 (32.5)	5 (22.7)	
Unknown *n* (%)	57 (50.9)	1 (10)	40 (50)	16 (72.7)	

*Electrical entry wound*					
Both hands *n* (%)	12 (10.7)	3 (30)	8 (10)	1 (4.5)	0.015^a^ 0.010^b^
Right hand *n* (%)	14 (12.5)	0	12 (15)	2 (9.1)	
Left hand *n* (%)	19 (16.9)	2 (20)	15 (18.8)	2 (9.1)	
Right arm *n* (%)	2 (1.8)	0	1 (1.2)	1 (4.5)	
Left arm *n* (%)	2 (1.8)	0	2 (2.6)	0	
Left foot *n* (%)	1 (0.9)	1 (10)	0	0	
Left leg *n* (%)	1 (0.9)	0	1 (1.2)	0	
Abdomen *n* (%)	1 (0.9)	1 (10)	0	0	
None *n* (%)	2 (1.8)	0	1 (1.2)	1 (4.5)	
Unknown *n* (%)	58 (51.8)	3 (30)	40 (50)	15 (68.3)	

*Electrical exit wound*					
Both feet *n* (%)	6 (5.3)	2 (20)	4 (5)	0	0.001^a^ 0.001^b^
Right hand *n* (%)	1 (0.9)	0	1 (1.3)	0	
Left hand *n* (%)	3 (2.7)	0	3 (3.6)	0	
Left hand and foot *n* (%)	2 (1.8)	1 (10)	1 (1.3)	0	
Right foot *n* (%)	2 (1.8)	2 (20)	0	0	
Right elbow *n* (%)	1 (0.9)	1 (10)	0	0	
Left foot *n* (%)	3 (2.7)	0	2 (2.5)	1 (4.5)	
Dorsal *n* (%)	2 (1.8)	1 (10)	1 (1.3)	0	
None *n* (%)	26 (23.2)	0	24 (30)	2 (9.1)	
Unknown *n* (%)	66 (58.9)	3 (30)	44 (55)	19 (86.4)	

^a^Analysis conducted with unknown variables for voltage status.

^b^Analysis conducted without unknown variables for voltage status.

**Table 3:** Factors affecting clinical features in electrical injuries.

Variables	All electrical injuries (*n* = 112)	High voltages (*n* = 10)	Low voltage (*n* = 80)	Unknown voltage (*n* = 22)	*p* value
*Creatinine level*					
Increased *n* (%)	5 (4.5)	3 (30)	2 (2.5)	0	0.002^a^ 0.003^b^
Decreased *n* (%)	18 (16.1)	1 (10)	14 (17.5)	3 (13.6)	
Normal *n* (%)	73 (65.2)	6 (60)	54 (67.5)	13 (59.1)	
Unknown *n* (%)	16 (14.3)	0	10 (12.5)	6 (27.3)	

*Sodium level*					
Decreased *n* (%)	6 (5.3)	1 (10)	4 (5)	1 (4.5)	0.122^a^ 0.425^b^
Normal *n* (%)	89 (79.5)	9 (90)	66 (82.5)	14 (63.6)	
Unknown *n* (%)	17 (15.2)	0	10 (12.5)	7 (31.8)	

*Potassium level*					
Increased *n* (%)	1 (0.9)	0	1 (1.3)	0	0.039^a^ 0.057^b^
Decreased *n* (%)	5 (4.5)	2 (20)	2 (2.5)	1 (4.5)	
Normal *n* (%)	89 (79.5)	8 (80)	67 (83.7)	14 (63.6)	
Unknown *n* (%)	17 (15.2)	0	10 (12.5)	7 (31.8)	

*Calcium level*					
Increased *n* (%)	3 (2.7)	0	3 (3.7)	0	0.028^a^ 0.051^b^
Decreased *n* (%)	8 (7.1)	3 (30)	5 (6.2)	0	
Normal *n* (%)	80 (71.4)	7 (70)	58 (72.5)	15 (68.2)	
Unknown *n* (%)	21 (18.7)	0	14 (17.5)	7 (31.8)	

*Troponin T level*					
Increased *n* (%)	57 (50.9)	6 (60)	45 (56.2)	6 (27.3)	0.072^a^ 0.345^b^
Normal *n* (%)	35 (31.2)	4 (40)	22 (27.6)	9 (40.9)	
Unknown *n* (%)	20 (17.9)	0	13 (16.2)	7 (31.8)	

*CK-MB level*					
Increased *n* (%)	25 (22.3)	7 (70)	13 (16.2)	5 (22.7)	0.001^a^ 0.001^b^
Normal *n* (%)	67 (59.8)	3 (30)	54 (67.5)	10 (45.5)	
Unknown *n* (%)	20 (17.9)	0	0	0	

*ECG*					
Normal *n* (%)	82 (73.2)	7 (70)	60 (75)	15 (68.2)	0.242^a^ 0.264^b^
Abnormal *n* (%)	16 (14.3)	3 (30)	11 (13.7)	2 (9.1)	
Unknown *n* (%)	14 (12.5)	0	9 (11.3)	5 (22.7)	

^a^Analysis conducted with unknown variables for voltage status.

^b^Analysis conducted without unknown variables for voltage status.

**Table 4:** Distribution of troponin T levels and ECG findings across voltage exposure groups.

Variables	All electrical injuries (*n* = 112)	220 V (*n* = 51)	360–750 V (*n* = 29)	> 1000 V (*n* = 10)	Unknown (*n* = 22)	*p* value
*Troponin level*						
Increased *n* (%)	57	29 (56.9%)	16 (55.2%)	6 (60.0%)	6 (27.3%)	0.041^a^ 0.258^b^
Normal *n* (%)	35	11 (21.6%)	11 (37.9%)	4 (40.0%)	9 (40.9%)	
Unknown *n* (%)	20	11 (21.6%)	2 (6.9%)	0 (0.0%)	7 (31.8%)	

*ECG status*						
Normal *n* (%)	16	6 (11.8%)	5 (17.2%)	3 (30.0%)	2 (9.1%)	0.417^a^ 0.422^b^
Abnormal *n* (%)	82	38 (74.5%)	22 (75.9%)	7 (70.0%)	15 (68.2%)	
Unknown *n* (%)	14	7 (13.7%)	2 (6.9%)	0 (0.0%)	5 (22.7%)	

^a^Analysis conducted with unknown variables for voltage status.

^b^Analysis conducted without unknown variables for voltage status.

**Table 5:** Relationship between ECG findings and troponin T levels.

Variables	All electrical injuries (*n* = 112)	Abnormal (*n* = 16)	Normal (*n* = 82)	Unknown (*n* = 14)	*p* value
*Troponin level*					
Increased *n* (%)	20	13 (22.8%)	44 (77.2%)	0 (0.0%)	0.001
Normal *n* (%)	57	2 (5.7%)	33 (94.3%)	0 (0.0%)	
Unknown *n* (%)	35	1 (5.0%)	5 (25.0%)	14 (70.0%)	

## Data Availability

The data that support the findings of this study are openly available in Mendeley Data at https://data.mendeley.com/datasets/c7jfmhd7py/1 [15].
